# Many Important Matters Decided

**Published:** 1921-02-12

**Authors:** 


### THE GENERAL NURSING COUNCIL.

### Many Important Matters Decided.

A meeting of the General Nursing Council for England and Wales was held at the Ministry of Health on Wednesday morning, February 2. The Chairman of the Council, Mr. J. C. Priestley, K.C., was in the chair, and those present were: Lady Hobhouse, the Hon. Mrs. Eustace Hills, Miss M. J. Tuke, Dr. E. W. Goodall, Dr. Bedford Pierce, Sir T. Jenner Verrall, Miss A. Cattell, Mr. T. Christian, Miss A. Coulton, Miss R. Cox-Davies, Miss A. Dowbiggin, Mrs. E. G. B. Fenwick, Miss A. Lloyd-Still, Miss M. MacCallum, Miss Isabel Macdonald, Miss E. Smith, Miss M. E. Sparshott, Miss E. C. Suiss, Miss S. E. Villiers, Miss C. Worsley, Miss C. Seymour Yapp, and Miss Riddell, the Registrar.

The minutes of the last meeting were agreed to, and the Chairman presented a letter from the Matron of the Stratford-on-Avon Hospital, which it was agreed to submit to the Education and Examination Committee for consideration. A letter was read from the Bradford Royal Infirmary Nurses' League, representing two hundred trained nurses, asking that special regulations should be made to protect nurses from overwork, but at the same time leaving them liberty of action to carry out their duties in the interests of their patients. The League deprecates legislation such as inclusion in the Forty-eight Hours Bill, believing that it may lower the status of trained nurses. On a suggestion that the matter should be referred to the Registration Committee, Mrs. Bedford Fenwick pointed out that that Committee does not deal with labour questions. It was decided by the Council to send a copy of its resolution on nurses' hours, and, on the proposal of Dr. Bedford Pierce, to ask the League for any suggestions to protect nurses from overwork.

A letter was submitted from the Public Health Department of Huddersfield inquiring, on behalf of the Medical Officer of Health, as to the constitution of the General Nursing Council—whether called into being by Act of Parliament or by Ministerial Order, or whether it is a voluntary body, and what its relations to the Ministry of Health are. The Chairman reported that a reply had been sent pointing out that the General Nursing Council is a statutory body appointed under the Nurses' Registration Act, 1919; that its members are twenty-five, appointed by the Privy Council, the Board of Education, and the Ministry of Health; that special branches of nursing are represented on the Council, general hospitals, Poor-law infirmaries, and children's, mental, fever, district, public health, and private nurses. To this letter a reply has been received from the Medical Officer of Health (Dr. S. G. Moore) that the Council's request that its schedule should be completed has been considered by the Sanitary Authority; it was felt that the labour involved would be considerable, and it was decided not to comply with the request.

### A Question of Principle.

The Council has received a letter from the Executive of the Central Council for District Nursing in London asking that the General Nursing Council should appoint a representative as a member of that Council for the ensuing three years. Mrs. Bedford Fenwick pointed out that the representation of a statutory body on an unincorporated body involves an important question of principle. The General Nursing Council is a semi-judicial body, and therefore it is undesirable it should be represented on nurses' organisations which have not the same statutory standing. In reply to her expressed desire to know the custom of the General Medical Council in similar circumstances, Sir T. Jenner Verrall said he did not know the general custom, but he felt fairly certain the General Medical Council has no representation on that particular body, which, however, does excellent work. Miss Cox-Davies seconded Mrs. Bedford Fenwick. She was of opinion that the Council should not agree to representation which might hamper its action very much. Miss Macdonald supported, and pointed out that acceptance would inevitably lead to the receipt of endless requests for representation on all sorts of bodies.

The resolution that the request should be declined was carried unanimously; it was agreed that the ground of refusal was that as a semi-judicial body it is inadvisable for the General Nursing Council to be associated with any other body dealing with nursing; at the same time, it was made clear that there is no lack of sympathy with the work of the Central Council for District Nursing.

### Hours of Employment Bill Again Discussed.

Mr. T. Christian, representing mental nurses, then proposed a resolution that nurses should come within the scope of the Ministry of Labour's Bill, except where a majority of the nurses in any distinct branch of the nursing profession decides to make application to be excluded from the provisions of the Bill, in which event only the branch of the profession making the application should be excluded. He regretted his resolution was not brought forward before Sir T. Jenner Verrall's resolution passed at the last Council (see The Hospital, December 18, page 274) was sent to the Minister of Health. Dr. Goodall here interposed an inquiry whether any answer had been received from the Minister of Health, and was informed that no answer had been received. Mr. Christian continued that the resolution passed by the Council disregards the wishes and desires of mental nurses and of a minority of nurses generally, that the Council's decision is harsh, arbitrary, and unconstitutional, and that on ethical grounds his resolution should be agreed to. Miss MacCallum, in seconding the resolution, said that, speaking for private nurses, she has not come across any who do not desire to come under the Bill, and that a meeting of the nurses belonging to the organisation, of which she is Hon. Secretary, passed a unanimous resolution desiring to come under the Ministry of Labour's Bill. On Miss Yapp's request for the number of nurses represented on that organisation Miss MacCallum said that there need be no difficulty in this, providing all other nurses' organisations put their figures of membership on the table. This was supported by Miss Cox-Davies, who said it would be an excellent thing to have the numbers. The Chairman pointed out, however, that the discussion of such matters was out of order; the subject before the meeting was Mr. Christian's resolution. Dr. Bedford Pierce queried if that resolution was in order; should not the previous resolution be first rescinded? The Chairman held that Mr. Christian's resolution was in essence a rescinding resolution, and asked if the Council were prepared to rescind. Miss Cattell remarked there is a great difference of opinion among the profession; and Miss Tuke pointed out that the resolution as it stands would break the solidarity of the nursing profession. Let the whole profession come under the Minister of Labour's Bill rather than have it divided. If the Council should rescind she would prefer to rescind in complete reversal of the policy laid down in Sir Jenner Verrall's resolution of the last meeting.

Dr. Goodall said he hoped the Council would not pass the resolution before it, though he had been in favour of nurses coming under the Minister of Labour's proposed Bill. But having deliberately made up its mind to ask another Minister to introduce a Bill—it being clear that nurses want to be under one of the Ministries in order that their hours may not be too long—it was extremely foolish within a few weeks to go to that Minister and tell him the Council has altered its mind. In the Minister of Labour's Bill there were clauses by which various branches of nurses might be excluded from the terms of the Bill if agreed between themselves and their employers; that provision was rejected with the rest of the proposal. Until the Council should hear from the Minister of Health in reply to its request he did not consider it desirable to touch the subject. Miss Macdonald said she was in sympathy with the spirit of the resolution, but she holds it to be a great mistake for the Council to consider economic questions. She suggested postponement of the resolution in the hope of receiving a reply from the Minister of Health. Sir T. Jenner Verrall protested against postponement. A body would be foolish, he said, to refuse to change its attitude if fresh circumstances have been brought forward to cause it to modify its view, but no fresh circumstances had been produced. He was all in favour of compromise, and if Mr. Christian's point had been brought forward earlier he would have been disposed to consider it, but at this stage there was no point in contracting out by sections. In his view it would be exceedingly unfortunate if the resolution were adopted. The conditions of nursing must differ in various kinds of nursing, but for the sake of getting uniformity and solidarity the minority must put up with something less or more.

On the resolution being put to the meeting it was lost by a large majority, only four voting for it.

Sick Children's Nurses.

Miss Worsley then proposed two resolutions substituting two fresh clauses in the draft rules, which have not yet been sanctioned. They concern the recognition by the Council for the purposes of its Registers, so far as existing nurses are concerned, of training in a general hospital for children approved by the Council, together with evidence of certain training in an approved general hospital or Poor-law infirmary. As the Rules have not yet received the sanction of the Minister of Health (though sent down to him on September 24 last) we do not give the text of the resolutions, which were carried with certain verbal alterations, Miss Yapp supporting and Miss Coulton seconding. For the same reason we do not report the lengthy discussion which took place on both these motions.

### The Registration Committee's Report.

Mrs. Bedford Fenwick, Chairman of the Registration Committee, presented her report. Four questions had been referred to the Committee by the Council on December 10—*viz.* (1) the standard of qualification for sick children's nurses; (2) the question of nurses trained in one country and practising in another; (3) the notification of other Councils of a nurse removed from the Register; and (4) jurisdiction in case of offences committed. The Committee's recommendations were presented, but in view of the fact that these matters had been discussed the previous day at a meeting with Dr. Addison, decision was held over pending the Minister's reply.

Mrs. Fenwick then submitted a specimen certificate of Registration for Existing Nurses and the Seal, which were approved, and the recommendation that they be adopted was seconded by Dr. Goodall, and carried unanimously. The estimate for 25,000 certificates (20,000 on the General Register and 5,000 on Supplementary Registers) is £175.

Two-Guinea Fee for Intermediate Nurses.

The fee for intermediate nurses (*i.e.*, those whose training is completed after November 1, 1919) after the term of grace, which lasts for two years after the rules come into operation, was then considered. The proposal to make this fee two guineas instead of one guinea as for existing nurses was questioned by Miss Yapp. Mrs. Fenwick stated that it is considered that privileges should terminate at the end of the term of grace, and the Chairman pointed out that the one-guinea fee is held to be too low, and that two guineas has been fixed on an economic basis. The recommendation was adopted.

The Committee next sought the sanction of the Council to draft rules for future nurses, and to provide for the election of nurse-members of the Council. [The present Council under the Act holds office for not less than two and not more than three years from the commencement of the Act, *i.e.*, November 1919.] It was pointed out that the work of the Education and Examination Committee has so progressed that this step is desirable. This recommendation was seconded by Sir T. Jenner Verrall, and carried unanimously.

Propaganda by the General Nursing Council.

The Council's authority to the Registration Committee was sought to compile and issue a Bulletin or small pamphlet giving exact information concerning the rules and regulations, training-schools for nurses, and the provisions of the Nurses' Registration Act. This was seconded by Miss Sparshott, and unanimously adopted. The recommendation that representatives of the Council should be sent to various examination centres to explain the system of examination and the method of election called forth from Sir T. Jenner Verrall the comment that the Bulletin should suffice; he doubted, if nurses would not read that, whether they would pay any attention to a representative, and he thought the expense might prove to be too great. Miss Sparshott suggested that the nurses in the different areas to which a representative was sent would pay the expense, as they do now in similar circumstances, and Sir T. Jenner Verrall then moved an amendment to the recommendation to the effect that the Council would send such representatives provided the expenses are paid. This was not seconded. Mrs. Fenwick urged the necessity to do everything to carry out the Act; she did not think it should be left to private associations to pay such expenses, and she pointed out the advantages of the spoken over the written word. Nurses have a right to know what the Council is doing, and to be instructed, and the money for representatives' visits would be money well spent. Miss Yapp seconded the recommendation, which was adopted, and the Chairman undertook that a rule should be framed to give the Council the necessary power, and that such rule should be brought before the next meeting.

Nurses' Views on the Uniform and Badge.

It was recommended by the Registration Committee that this question should be thrown open to the profession for an expression of individual opinion as to the sort of uniform desired, and that a *communiqué* should be drafted by the Registration Committee and sent out through the Press. The views of nurses should also be invited with regard to the badge. This was seconded by Miss Cattell, and adopted. It was pointed out by Miss Villiers, and agreed, that the wearing of uniform or badge should be optional.

### The Education and Examination Committee.

The report of this Committee was presented by Miss Lloyd-Still, its Chairman. The recommendations include the following: That a syllabus of nursing education shall be circulated to hospitals and Poor-law infirmaries for their guidance in training nurses. This was seconded by Miss Cattell, and adopted. That the first State examinations shall be held in July and October 1923, and in January and April 1924. These examinations will be voluntary. After April 1924 those who desire to register will require to pass a State examination, the first of which will be held in July 1924, and subsequent examinations in October, January, April, and July in each year. Fourteen places were recommended as suitable centres for examination, others to be added if necessary—*viz.*, London, Birmingham, Bristol, Leeds, Liverpool, Manchester, Newcastle-on-Tyne, Cardiff, Carlisle, Norwich, Nottingham, Portsmouth, Exeter, and Sheffield. These recommendations were adopted, Miss Lloyd-Still pointing out, in reply to Dr. Bedford Pierce's inquiry whether it was not premature at this stage to fix examination centres, that it is desirable to get a basis on which to work.

A recommendation to hold an informal conference as to future training with matrons and teachers of nursing was adopted. It was pointed out that it is highly desirable to enlist the sympathy of every one interested in the teaching of nurses, so that all sections of nursing shall be in some way brought into the curriculum. The question of reciprocal curricula also requires wisdom in its handling.

### The Finance Committee.

Sir T. Jenner Verrall presented the report of this Committee. The principal item was the future headquarters of the General Nursing Council at York Gate. The lease is now being prepared, and the seal of the Council will be affixed in due course. A Sub-Committee is to procure estimates for furnishing with a sum of £1,500 in mind. The Gas Light and Coke Co. has undertaken to fit gas stoves, etc., for half the amount which would in the ordinary course be charged. This concession, the Chairman stated, is because the work is done for a Council connected with nurses.

The next meeting of the Council will take place after the receipt of the reply of the Minister of Health to matters submitted to him.

